# COVID-19: The effects of perceived organizational justice, job engagement, and perceived job alternatives on turnover intention among frontline nurses

**DOI:** 10.3389/fpsyg.2022.920274

**Published:** 2022-09-06

**Authors:** Lulin Zhou, Arielle Doris Tetgoum Kachie, Xinglong Xu, Prince Ewudzie Quansah, Thomas Martial Epalle, Sabina Ampon-Wireko, Edmund Nana Kwame Nkrumah

**Affiliations:** ^1^Centre for Medical Insurance, Hospital Management and Health Policy Research, School of Management, Jiangsu University, Zhenjiang, China; ^2^School of Management, Jiangsu University, Zhenjiang, China; ^3^Department of Computer Engineering, School of International Business, Zhejiang International Studies University, Hangzhou, China; ^4^School of Environment and Safety Engineering, Jiangsu University, Zhenjiang, China

**Keywords:** organizational justice, job engagement, perceived job alternatives, turnover intention, COVID-19, frontline nurses

## Abstract

Nurses’ turnover intention has become a concern for medical institutions because nurses are more needed than ever under the prevalence of COVID-19. This research sought to investigate the effects of the four dimensions of organizational justice on COVID-19 frontline nurses’ turnover intention through the mediating role of job engagement. We also tested the extent to which perceived job alternatives could moderate the relationship between job engagement and turnover intention. This descriptive cross-sectional study used an online survey to collect data from 650 frontline nurses working in appointed hospitals in Jiangsu province, China. Hierarchical regression was used to analyze the hypothesized relationships. Findings revealed that all organizational justice components significantly influenced job engagement and turnover intention. Job engagement also significantly affected nurses’ turnover intention and mediated the relationships between organizational justice components and turnover intention. Besides, perceived job alternatives moderated the relationships between job engagement and turnover intention. The implications of this study include demonstrating that healthcare authorities should respect human rights through effective organizational justice as this approach could encourage nurses to appreciate their job and be more devoted to staying and achieving their institutional duties, especially under challenging circumstances.

## Introduction

Nurses’ role in any community is vital, as they work 24/7 to provide patients with quality care services. Since the end of 2019, when the COVID-19 pandemic tragically attacked the world, frontline nurses have shown bravery and courage to save people’s lives. COVID-19 has been a shock for the world and its population, and the challenges to global health are still evident (World Health Organization, 2021). Nursing personnel was no less involved in China, where the pandemic erupted. Experienced, newly licensed, and even volunteer student nurses had to put efforts together considering the emergency of the situation (Zhang W. et al., 2020; Zhang Y. et al., 2020). Older nurses had to put their experience and courage forward. Also, newly registered nurses had to be more determined than ever to embrace their new work environment despite the pandemic. Volunteer student nurses had to acknowledge the challenges the work environment could bring into their lives. The conditions were such that frontline nurses had to simultaneously protect their lives and be on duty (Cai et al., 2020; Thorne, 2020).

According to Zhou et al. (2021) and Obeng et al. (2021), one can easily feel demotivated to continue working under conditions that threaten their existence. Recent research showed that COVID-19 increased turnover intention among healthcare personnel. Typical examples happened in the Philippines (Labrague and de Los Santos, 2020), Peru (Yáñez et al., 2020), Pakistan (Irshad et al., 2021), and China as well (Mo et al., 2020; Nie et al., 2020; Zhang W. et al., 2020; Hou et al., 2021).

Regardless of its old age and the significant number of academic research papers dealing with the topic, turnover intention remains a dynamic field of research, especially with the advancement of new managerial techniques for workers’ retention, labor market dynamism, the development of technology, new research methods, and constant environmental changes. In many cases, turnover intention happens when people’s job gives them more dissatisfaction, anxiety, and fear than happiness (Jung et al., 2021). According to Chen et al. (2022), organizational justice is one possible factor that tends to create satisfaction or dissatisfaction, serenity or anxiety, happiness or melancholy, and courage or fear in many organizations, including the healthcare sector. It has also been linked to turnover in previous studies (Suifan et al., 2017; Hussain and Khan, 2019; Cao et al., 2020; Mengstie, 2020).

Colquitt et al. (2005) defined organizational justice as how workers feel about their company’s impartiality or fairness. It has four dimensions: distributive justice, procedural justice, interpersonal justice, and information justice (Nojani et al., 2012; Colquitt et al., 2013; Mengstie, 2020). Studies (e.g., Cao et al., 2020 and Mengstie, 2020) that examined the relationship between organizational justice and turnover intention analyzed these four dimensions as a composite score of organization justice, making it unclear whether each dimension’s predictive effects on turnover intention differ. The current study will address this literature gap by examining the predictive capacity of the four dimensions of organizational justice on turnover intention among frontline nurses, especially in COVID-19. Frontline nurses constitute an appropriate sample to explore how the different aspects of organizational justice influence their turnover intention in such a demanding environment. Such a study might be relevant to reducing nurses’ turnover intention and, thus, their actual turnover. However, it may be complete if we know how (the influencing mechanism through which) these organizational justice components could directly or indirectly influence turnover intentions. Job engagement has been proposed in previous studies (e.g., Park and Johnson, 2019; Cao et al., 2020; Jung et al., 2021; and Chen et al., 2022) to mediate the relationship between turnover intention and its antecedent significantly.

Job engagement refers to a positive, meaningful, and enthusiastic attitude employees show to their organization when they like their job (Schaufeli et al., 2006). It can demotivate employees from leaving their current jobs (Moo and Sung, 2017; Zhang et al., 2018). Thus, organizations that create a climate that increases job engagement are likely to benefit from lower turnover than organizations that do not (Jung et al., 2021). Therefore, analyzing job engagement as a mediator between the nurses’ perception of justice and turnover intention will contribute to the literature. Despite the mediating capacity of job engagement on turnover intention and other antecedents, it is relevant to examine the conditions under which the impact of job engagement on turnover intention could either be strengthened or weakened. Alternative job opportunities (Dohlman et al., 2019) may be possible to provide conditions that can buffer the impact of work engagement on turnover intention.

Perceived job alternative refers to workers’ interest in the external labor market and their awareness of other available jobs (Sender et al., 2021). It can affect employees’ engagement as it increases one’s enthusiasm for their job (Dohlman et al., 2019). The assurance of getting a new job may also involve the cognitive aspect of staying or not in that job (Hom et al., 2012). Though precedent research demonstrated how the perception of alternative job opportunities might influence employees’ job attitudes and their movement desirability (Li et al., 2016), they failed to investigate whether it moderates the relationship between workers’ engagement and turnover intention. Thus, exploring such intervention is indispensable.

Also, several studies have proposed various theories to explain the relationships between turnover intention and its antecedents. There is the equity theory (Cao et al., 2020), the job demands-resources model (Carlson et al., 2017; Scanlan and Still, 2019), the conservation of resources theory (Jin et al., 2016), and the effort-reward-imbalance model (Derycke et al., 2011; Zeng et al., 2021) among others. This study employs social exchange theory (SET; Cropanzano and Mitchell, 2005) to espouse the linkages between organizational justice, job engagement, alternative job opportunities, and turnover intention.

The social exchange theory’s core theoretical and empirical aspects rest on reciprocity, social networks, fairness, solidarity, and social cohesion (Cook, 2015). The theory supposes that people’s social behaviors result from evaluating the benefits and costs during an exchange process. Individuals perform a behavior when they are assured that the resulting reward will at least be equal to or exceed the costs. Conversely, individuals will restrain from performing a behavior when inputs are more than outcomes and could even start looking at other options (Bies and Moag, 1986; Colquitt, 2001). From this viewpoint, medical industries’ exemplary implementation of organizational justice may enhance workers’ resources such as engagement, lower negative job attitudes such as turnover intention, and eradicate alternative job search behavior.

**Figure 1:**
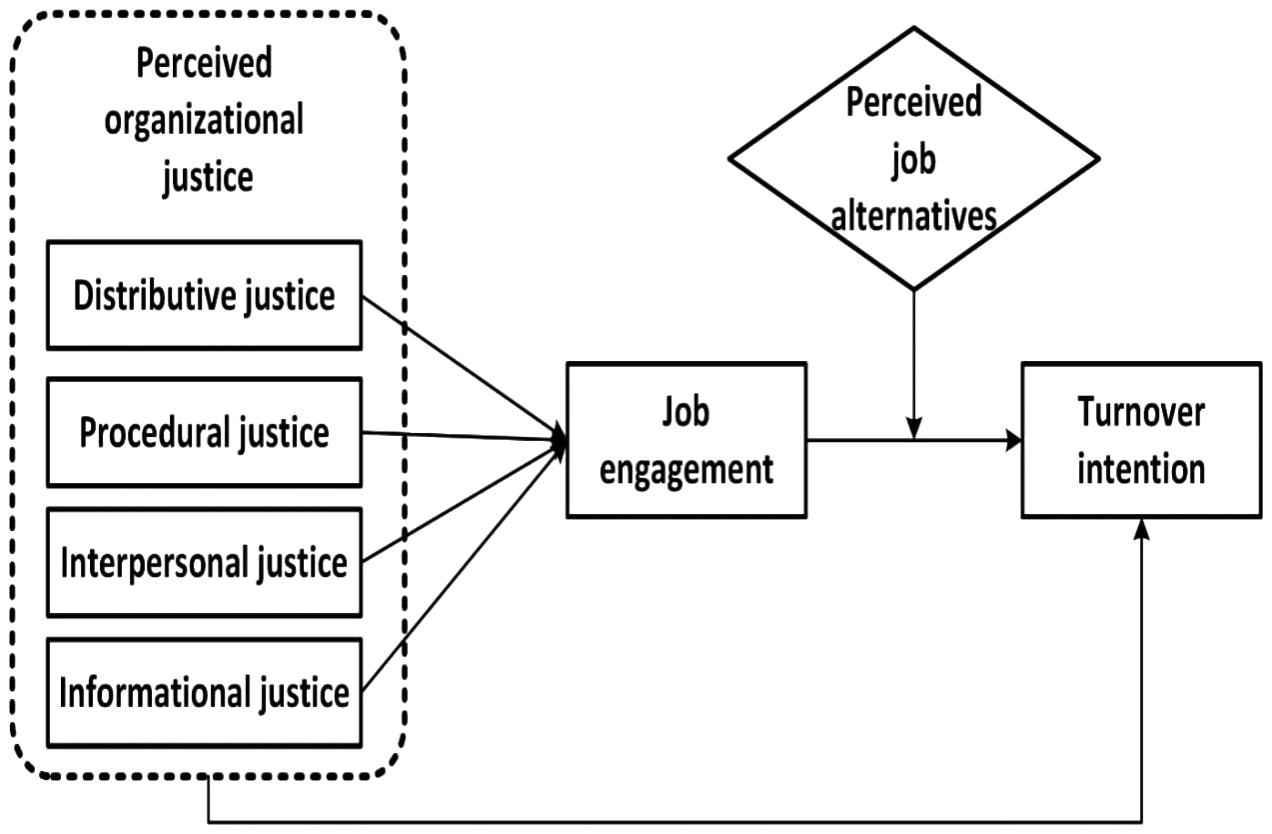
Proposed hypothesized model.

Thus, this study assumes that the pandemic aggressivity could affect nurses’ perception of justice in their workplace, influencing their engagement and willingness to continue in that profession or pushing them to find alternative jobs. Otherwise, despite the difficulties, they could also, with the appropriate resources, find themselves valuable enough for the current time and get more engaged to help the world go back to a healthier place (Thorne, 2020; Zhang Y. et al., 2020). Following this trend, this study aimed to explore organizational justice’s impact on turnover intention through job engagement among frontline nurses during the COVID-19 pandemic. Additionally, the moderating role of perceived job alternatives on job engagement and turnover intention relationship will be ascertained. The study also presents a conceptual framework (Figure 1) that espouses the linkages among the various variables under investigation.

## Literature review and hypotheses formulation

### Turnover intention

Turnover intention has been defined as an employee’s perception of the likelihood of leaving their current job or forfeiting their present position in an organization (Meyer et al., 1993; Tao et al., 2018). As a precursor of actual turnover (which is among the leading cause of the nursing shortage), the turnover intention has been addressed widely in the nursing literature in other parts of the world (Al Sabei et al., 2020; Bautista et al., 2020), as in China (Wang and Wang, 2020; Zhou et al., 2021). Damages to the medical organizations and employees’ lives make it continue to be a subject of growing interest, especially after the World Health Organization projection of about 12.9 million nurses shortage in the world by the end of 2035 (Marć et al., 2019). With the current COVID-19 pandemic, this projection could be more significant.

The COVID-19 pandemic has turned the world upside down, and frontline nurses’ lives have been impacted in many ways. Caring for COVID-19 patients has affected their mental health, wellbeing, and family life (Mo et al., 2020; Pappa et al., 2020; Wu et al., 2020). They had to deal with and quickly adapt to constantly changing protocols and policies and a patient load augmentation (Labrague and de Los Santos, 2020). Additionally, they have been exposed to discrimination and isolation (Al Sabei et al., 2020; Nie et al., 2020). With the experience of the previous sanitary crisis (SARS or Serious Acute Respiratory Syndrome) the country went through in 2002/2003, Chinese nurses knew how someone’s life could be affected (Catton, 2020). Evidence supports that the unexpected changing conditions made nurses more vulnerable and susceptible to quitting; they were highly concerned about their personal and family welfare since they had to balance facing a deadly virus and their ethical duty (Maben and Bridges, 2020). As He et al. (2020) reported, the prevalence of turnover intention before COVID-19 was already high at 30.4% among Chinese health personnel. It increased by 10.1% during COVID-19 (Hou et al., 2021). Though a plethora of research has assessed the factors influencing nursing turnover intention, it is still essential to consider the combination of other fundamentals affecting it since attitudes are contextual and are subject to change, and their understanding seems complex.

### Perceived organizational justice and job engagement and turnover intention

The concept of organizational justice has been increasingly given a universal concern based on its little emphasis on human rights (Topbaş et al., 2019). It has gained more consideration in the Western culture throughout the years; however, the non-Western world still needs more evidence on how its workers perceive it (Fodchuk, 2009; Cao et al., 2020). It is important to note that the perception of human rights has evolved with time and is also influenced by the differences in cultural backgrounds and levels of development (Kausikan, 1996). For example, taking someone’s right to life (committing murder) is a gross violation of human rights and should be sentenced accordingly in any civilized world. Still, in many aspects of human rights, the East, compared to the West, considers human rights as a process rather than specific outcomes (Seokwoo and Hee, 2020). The Eastern human rights idea results from historical developments which vary with liberal frameworks and democratic institutions. Their practice of human rights differs from the country and is integrated into people’s everyday life. It is closely related to religious beliefs, historical background, social conditions and values (Prakash and Murarka, 2021).

Organizational justice is perceived differently across cultures as a psychological construct since it expresses workers’ judgment of fairness or righteousness in their organizations (Quezada-Abad et al., 2019). Then, it can significantly influence organizational behaviors and outcomes (Colquitt, 2001). Evidence supports that perceived justice is positively associated with organizational behaviors such as job engagement (Cao et al., 2020; Suifan et al., 2020), trust (Bidarian and Jafari, 2012; AL-Abrrow et al., 2013), organizational citizenship behaviors (Chang, 2014; Sulander et al., 2016), organizational commitment (Ohana, 2014; Fardid et al., 2018), job satisfaction (Cassar and Buttigieg, 2015; Fardid et al., 2018), and negatively with turnover intention and other negative attitudes (Colquitt et al., 2013; Proost et al., 2015; Terzioglu et al., 2016) across various settings. Employees’ perception of injustice can predispose them to experience burnout; contrariwise, their engagement is enhanced when they think they are treated fairly by their institution. They want to stay and give the best of themselves (Maslach et al., 2001).

According to Colquitt (2001) and Colquitt et al. (2013), organizational justice is a multifaceted concept that includes four dimensions, distributive justice, procedural justice, interpersonal justice, and informational justice. Distributive justice has to do with fairness in outcomes’ distribution, like salary, promotion, or rewards. Workers try to evaluate the balance between their outputs and inputs. Here, employees’ cognitive, affective, and behavioral reactions are related to their judgment of how outcomes are distributed (Cropanzano et al., 2007). Procedural justice refers to ethics’ consistency, accuracy, and respect during decision-making and outcomes distribution (Leventhal, 1980; Quezada-Abad et al., 2019). It influences employees’ consideration for their organization and attitudes to stick with the company’s best interests. Interpersonal justice expresses how the authority enacts procedures or distributes outcomes with respect and dignity. When their organization appropriately treats workers, it enhances other good behaviors and willingness to comply with decisions (Bies and Moag, 1986; Colquitt, 2001). Informational justice deals with truth and adequacy in sharing information or implementing procedures. In providing information, the clarifications and justifications help elucidate any feeling of injustice and prevent adverse reactions among the personnel (Colquitt et al., 2013).

Drawing on the social exchange theory (SET; Cropanzano and Mitchell, 2005), organizations that can fulfill the various elements of organizational justice such that employees can perceive their dispensation as fair or equitable can persuade employees to reciprocate through devotion. Highly devoted employees show higher enthusiasm for work engagement than less devoted employees (Suifan et al., 2020). Therefore, we rely on SET and other related studies (e.g., Colquitt et al., 2013; Chen and Jin, 2014; Cao et al., 2020; and Chen et al., 2022) to propose the following hypotheses:

*H1*: Organizational justice will have a significant negative influence on turnover intention.

*H1a*: Distributive justice will have a significant negative influence on turnover intention.

*H1b*: Procedural justice will have a significant negative influence on turnover intention.

*H1c*: Interpersonal justice will have a significant negative influence on turnover intention.

*H1d*: Informational justice will have a significant negative influence on turnover intention.

*H2*: Organizational justice will significantly and positively affect nurses’ job engagement.

*H2a*: Distributive justice will significantly and positively affect nurses’ job engagement.

*H2b*: Procedural justice will significantly and positively affect nurses’ job engagement.

*H2c*: Interpersonal justice will significantly and positively affect nurses’ job engagement.

*H2d*: Informational justice will significantly and positively affect nurses’ job engagement.

### Job engagement and turnover intention

When employees like their job, one of the positive, enthusiastic responses they give to their organization is engagement (Ghosh et al., 2014). Therefore, it is a vital factor an organization can rely on to boost its productivity and profit and reduce employee turnover (Bin, 2016). According to Schaufeli et al. (2006), job engagement encapsulates vigor, absorption, and dedication, implying that people use their energy to immerse in and fulfill their life-driven purpose, which is their job. It has been related to many antecedents and consequences (Al-Tit and Hunitie, 2015). Positive outcomes related to workers’ engagement include satisfaction (Bin, 2016; Pieters, 2018), commitment (Al-Tit and Hunitie, 2015), good performance (Bin, 2016; Terzioglu et al., 2016), low intent to quit, and other positive behaviors (Bakker et al., 2011; Gupta et al., 2019). From the lenses of the job demands-resources (JD-R) model, the firm’s physical, social and psychological aspects are required resources that can empower workers’ intellectual and emotional association with it and lower their withdrawal behaviors (Bakker and Demerouti, 2007). Also, drawing on social exchange theory (SET; Cropanzano and Mitchell, 2005), employees feel more connected to their company, work hard, and encourage colleagues to do the same when higher engagement occurs. SET theorists (e.g., Agarwal, 2014; Ancarani et al., 2018; and Yin, 2018) highlight that promoting job engagement creates a validation environment for employees to function. Employees who feel validated show great enthusiasm, stay longer, and work for their organizations through reciprocity (Juhdi et al., 2013). Several other studies (e.g., Edwards-Dandridge, 2019; Cao et al., 2020; and Zhang X. et al., 2020) also found work engagement to mitigate one’s desire to leave their work voluntarily. Therefore, from the perspective of social exchange theory and other related literature, the study hypothesizes that:

*H3*: Job engagement has a significant negative influence on frontline nurses’ turnover intention

### Job engagement as a mediator

Previous studies have demonstrated the mediating capacity of job engagement between its causing variables and those it impacts in various settings. For instance, job engagement has been shown to mediate significantly, among others, the relationships between organizational justice and job performance among airline employees in Jordan (Suifan et al., 2020), between job and personal resources, and turnover intention among female nurses in Iran (Shahpouri et al., 2016), between organizational support and intention to leave among healthcare employees in Turkey (Baş and Çınar, 2021), between job insecurity and safety behavior among enterprises employees (Zhang et al., 2021), or between job characteristics and job satisfaction among public banks workers in India (Rai and Maheshwari, 2021). From the literature review, organizational justice can influence job engagement (Zhu et al., 2015; Park et al., 2016; Özer et al., 2017) and turnover intention (Agbaeze et al., 2018; Perreira et al., 2018; Huang et al., 2019). Also, studies like Park and Johnson (2019), Edwards-Dandridge et al. (2020), and Quek et al. (2021) have demonstrated that job engagement significantly influences turnover intention. Therefore, drawing on social exchange theory (SET), we believe that employees who fit in their work due to acceptable organization justice may be better engaged and show lower motivation to leave. For instance, the healthcare sector consists of inter-related activities and resources, including compensation, promotions, disciplinary procedures, performance appraisal, training, holidays, leave with pay, etc. From SET perspectives (Williamson and Williams, 2011), nurses who perceive inequality in distributing these resources may feel injustice. They may exhibit their displeasure in the form of lower job engagement (Chênevert et al., 2013). In contrast, nurses may feel justice or fairly treated if they perceive the equitable distribution of organizational resources. They may be motivated and inspired by the actions of their organization. Such nurses may reciprocate their organizations’ kind gestures through higher job engagement (Yin, 2018), which may reduce turnover intention (Memon et al., 2014; Edwards-Dandridge, 2019). From this background, it is expected that organizational justice increases work engagement and work engagement may reduce turnover intentions. Therefore, relying on the social exchange theory, we hypothesized that:

*H4*: Job engagement will significantly mediate the relationship between organizational justice and frontline nurses’ turnover intention.

*H4a*: Job engagement will significantly mediate the relationship between distributive justice and frontline nurses’ turnover intention.

*H4b*: Job engagement will significantly mediate the relationship between procedural justice and frontline nurses’ turnover intention.

*H4c*: Job engagement will significantly mediate the relationship between interpersonal justice and frontline nurses’ turnover intention.

*H4d*: Job engagement will significantly mediate the relationship between informational justice and frontline nurses’ turnover intention.

### Perceived job alternatives as a moderator

As human beings, workers always strive to look at what is best for themselves (Tay and Diener, 2011; Taormina and Gao, 2013). Their perception of their profitability and the availability of opportunities in the labor market influence their working behaviors and attitudes (Dohlman et al., 2019). Employees’ turnover intentions have been associated with alternative job opportunities. It is stated that turnover cognitions are prompted in employees who show low job attitudes or have job search behavior (Hom et al., 2012). For example, Sender et al. (2021) highlighted that workers with high turnover intentions had many job opportunities and presented severe deviant behaviors. The turnover intention was similarly predicted by perceived job alternatives in a study by Wossen and Alemu (2018), where personnel with a higher turnover intention had an increased perception of job availability outside the company.

Prior research has also established how perceived job alternatives can moderate and alter a bivariate causal relationship by strengthening or weakening it. Because of previous antecedents, withdrawal behaviors from workers may be observed depending on their perceived level of alternative jobs. In a study by Robert and Vandenberghe (2017), alternative job opportunities moderated the relationship between openness to experience and intention to quit. Another study by Van Hootegem et al. (2019) similarly observed that perceived job opportunities played a moderating role between job insecurity and the willingness to undertake training. Within a correlation framework as a third variable, perceived job alternative has been shown to affect the zero-order correlation between two other variables (Baron and Kenny, 1986).

Workers with a high perception of alternative job opportunities show less positive job attitudes such as job satisfaction, commitment, or job engagement and are most likely to leave (Li et al., 2016; Dohlman et al., 2019). Furthermore, these positive job attitudes negatively influence turnover intention, making perceived job alternatives satisfy the moderation criterion. The social exchange theory also explains that the relationship evolves with time and reaches different stages during an exchange process. At the beginning of the relationship, individuals may ignore the social exchange balance but start contrasting the costs and benefits with time. If they estimate the results are not equitable, they may start evaluating the alternatives, which may eventually affect the nature of the relationship (Emerson, 1976; Lambe et al., 2001). From this premise and based on the fact that limited research has examined how perceived job alternatives could alter the direction between job engagement and turnover intention; we then suggest the following hypothesis:

*H5*: Perceived job alternatives will moderate the relationship between job engagement and turnover intention.

## Materials and methods

### Study design and settings

This research used a descriptive and cross-sectional study design based on STROBE guidelines, using an online questionnaire survey to collect data. Data were collected from frontline nurses working in tertiary hospitals across Jiangsu province in China. Third-level hospitals are big-sized hospitals with more than 500 beds and equipped with advanced technologies offering high-quality, specialized, and comprehensive medical care, with possibilities for medical education and research, and are found in large cities (Zhang et al., 2013; La Forgia and Yip, 2017).

A purposive and random sampling technique was used to select 14 tertiary hospitals among the 28 designated to treat pneumonia caused by the novel coronavirus in Jiangsu Province. For a better representation, one of the two hospitals was selected in each of the 13 prefecture-level cities of Jiangsu Province and the 14th among the two at the provincial level.

### Participants

Registered nurses formally or contract employed, on duty during the survey, who have been working for at least 6 months in their actual work unit and who have been directly involved in taking care of patients with coronavirus were eligible to participate in this study. We adopted a sample-to-variable ratio for the sample size determination, presented as the N: p ratio, where N and p are the numbers of participants and items, respectively (Chatterjee and Hadi, 2015; Sprent and Smeeton, 2016). More specifically, a 10: 1 ratio was used (as proposed by Cattell (1978), and cited by Kline (2014)) to determine sample size, indicating that for the 31 items of the survey, a total number of 310 participants would be sufficient. The online survey was then conveniently sent to 650 frontline nurses (approximatively the double amount required to ensure greater participation), of which 576 submitted the questionnaire, making a response rate of 88.6%.

### Data collection

Data collection was done under the approval of various hospital authorities between September and October 2021, a few months after the outbreak in Nanjing city. An online data collection approach was adopted due to the pandemic’s restrictions. Hospitals’ administrators helped share the questionnaire link with nurses working in different hospitals through nurses’ supervisors. Nurses’ supervisors then forwarded the link to their networking groups to make it accessible, mainly through WeChat, a well-liked mobile application in China. The questionnaire included four standardized scales and socio-demographic characteristics such as gender, age, education level, marital status, employment status, salary, and years of experience. To manage common method biases and also deal with invalid responses appropriately, we designed the questionnaires in a way that could have the tenacity to prevent issues related to invalid responses. It was designed in a manner that made all the questions compulsory. Hence, failing to respond to one question would not allow one to submit the survey. Therefore all submitted online responses by the respondents were filled without errors. The respondents’ ability to fill the online surveys appropriately could emanate from the fact that nurses were informed in the introductory text that preceded the questionnaire that they should answer all the questions if they choose to participate. Also, prior to issuing the QR-Code and online link containing the online survey, the authors sought clearance from the authorities of these hospitals. We explained to them the objectives of the study. After receiving the approval, we held short meetings with the various frontline nurses’ supervisors, explained to them the aim of our research, and requested them to encourage nurses under their supervision to participate in the online survey issued to them by their administrators in the form of either QR-Code or an online link. We assured them of the utmost anonymity and privacy of their responses. We informed the respondents that their responses would only be used for academic exercise and that their identity would not be disclosed to anyone in any way or any form. We also requested them to fill out the online survey sent to them by their administrators with all honesty and sincerity. We reminded them that there was no wrong or correct answer and that they could choose the most appropriate response that addresses or fits their current situation. In all our meetings with the respondents, we highlighted that participation was voluntary. Moreover, those who choose to participate could decline anytime they deem necessary. Employing these approaches helped manage biases and invalid responses.

### Measures

Since the available versions of the scales were in English (see Appendix A), their items were reverse translated into Chinese. Back-translation and standard blind translation were adopted for this purpose (Brislin, 1986), and two expert translators achieved this to ensure the consistency and the equivalence of the meanings. The first translated from English to Chinese, and the second did the reverse translation from Chinese back to English as recommended by Hambleton (2005). Two professors in the research team and four doctoral students helped judge and certify the content validity of those same items. A pilot study was conducted on 45 nurses before the survey was released online. The pilot testing was proper to refine the items and ensure no ambiguities with the meanings. Fortunately, no significant issues were pointed out by nurses. Besides, nurses enrolled for that were not included in the final analysis.

### Perceived organizational justice

Perceived organizational justice was measured using one of the most widely used justice measures proposed by Colquitt (2001). The scale has 20 items distributed among four dimensions: distributive justice (4 items), procedural justice (7 items), interpersonal justice (4 items), and informational justice (5 items). The constructs showed good internal consistency and reliability, with Cronbach’s alphas ranging from 0.78 to 0.93 (Colquitt, 2001; Omar et al., 2018). All items were measured on a five-point Likert scale ranging from 1 (strongly disagree) to 5 (strongly agree), with higher scores indicating a stronger perception of justice. It is a well-validated scale, used in several other studies and across various settings (Díaz-Gracia et al., 2014; Omar et al., 2018). The overall instrument Cronbach’s alpha coefficient in this study was 0.746, and that of the different constructs was 0.936, 0.922, 0.871, and 0.889 for distributive justice, procedural justice, interpersonal justice, and informational justice, respectively.

### Job engagement

Job engagement was assessed using five items of the job engagement scale taken from the study of Jung et al. (2021), which they adapted from Schaufeli et al. (2002) and Schaufeli et al. (2006). Responses were rated on a seven-point Likert scale from 1 (never) to 7 (always), and higher scores showed greater engagement. The Cronbach’s overall alpha of 0.97 indicated a high internal consistency (Jung et al., 2021). The reliability result of the items for this work was 0,877, judged acceptable.

### Perceived job alternatives

The perception of nurses’ alternative job opportunities was analyzed using three items, rated on a five-point Likert scale, from 1 (strongly disagree) to 5 (strongly agree). An increase in the score indicated an increased perception of job alternatives. The scale was picked from the work of Mushtaq et al. (2014), which they adapted from Mowday et al. (1984). Their study reported a good predictive validity and reliability of the scale (Cronbach alpha = 0.73; Mushtaq et al., 2014), and in this study, the internal consistency was also good, being 0.941.

### Turnover intention

The three-item turnover intention scale developed by Singh et al. (1996) was adopted for this study. Its predictive validity and reliability were initially 0.89 and, in the current research, 0.831. Answers were measured on a seven-point Likert scale ranging from 1 (never) to 7 (always). A higher score suggested an increase in the intention to leave.

### Data analysis

SPSS version 26 was used to analyze the descriptive aspects of the data and to perform exploratory factor analysis (EFA). The EFA helped ensure that the survey items were loaded under their predicted components. Besides, AMOS version 22 was used to perform confirmatory factor analysis (CFA). The CFA was conducted to provide additional robust support to the data set. We also used hierarchical regression analysis in SPSS to test the various hypothesized relationships.

## Results

### Respondents’ characteristics

Of the 576 responses received, 72.7% (419) were female nurses, and 27.3% (157) were males. Their ages were distributed among 20–30 (40.3%), 31–40 (37.3%), and 41–50 (22.4%) years old. Also, 354 (61.5%) graduated from the university, and 222 (38.5%) studied in a vocational school. There were 239 (41.5%) formally employed nurses, while 337 (58.5%) worked on a contract basis. Moreover, 451 (78.3%) nurses were married, and 125 (21.7%) were not. Regarding their salary, about 385 (66.8%) nurses admitted receiving a monthly salary greater than 5,000 RMB, 83 (14.4%) and 79 (13.7%) said their monthly salary range is between 4,001–5,000 RMB, and 3,001–4,000 RMB, respectively, the rest of 29 (5%) said they receive less than 3,000 RMB a month. Among the participants, 271 (47%) have been working for more than ten (10) years, 166 (28.8%) for 6–10 years, 126 (21.9%) for 1–5 years, and only 13 (2.3%) have worked for less than a year.

### Common method variance

Podsakoff et al. (2003) indicate that common method biases could exist if data is collected from single sources. Therefore, this current study employed procedural and statistical approaches recommended in previous studies (Podsakoff et al., 2003; Chang et al., 2010; Jordan and Troth, 2020; and Rodríguez-Ardura and Meseguer-Artola, 2020) in handling issues related to a common method bias. Procedurally, we obtained permission from appropriate authorities, guaranteed respondents’ anonymity and confidentiality, and encouraged respondents to respond to the questionnaire items honestly since there was no right or wrong answer. We statistically checked for common method bias by performing Harman’s single factor test with exploratory factor analysis in SPSS version 26 software. The results showed that a single factor could explain only 26.251% of the total variance. Hence, a single could not account for more than 50% of the total variance, suggesting that our data did not suffer from common method biases.

### Reliability and validity of the scales

The exploratory factor analysis (EFA) results showed that all items had good loadings above 0.50. The recorded values of Kaiser–Meyer–Olkin measure of sampling adequacy (KMO-MSA; 0.886) and Bartlett’s test of sphericity (BTS; *X*^2^ = 12,518.172; df = 465; *p* < 0.001) were within acceptable thresholds.

As shown in Table 1, the CFA’s factors loadings revealed that the factor loadings for the variables were greater than the 0.50 suggested thresholds (0.669–0.999). Each factor loading had a significant value of *p* < 0.001.

**Table 1:** CFA factor loadings, reliability, and validity results.

Variables	Codes	Factor loadings	SE	CR	*α*	CR	AVE
Procedural justice (PJ)	PJ1	0.901			0.922	0.922	0.63
	PJ2	0.815	0.034	26.229			
	PJ7	0.789	0.035	24.695			
	PJ3	0.781	0.035	24.25			
	PJ5	0.748	0.037	22.504			
	PJ4	0.772	0.036	23.79			
	PJ6	0.741	0.037	22.167			
Informational justice (IJ)	IF4	0.995			0.889	0.894	0.633
	IF5	0.746	0.032	25.165			
	IF3	0.797	0.03	29.047			
	IF2	0.684	0.033	21.435			
	IF1	0.717	0.032	23.317			
Job engagement (JE)	JE1	0.860			0.877	0.879	0.594
	JE4	0.793	0.042	22.211			
	JE2	0.758	0.044	20.836			
	JE5	0.759	0.043	20.875			
	JE3	0.669	0.047	17.559			
Distributive justice (DJ)	DJ4	0.999			0.936	0.938	0.791
	DJ1	0.853	0.022	36.973			
	DJ3	0.840	0.024	35.257			
	DJ2	0.855	0.022	37.332			
Interpersonal justice (IT)	IT1	0.965			0.871	0.879	0.649
	IT4	0.771	0.033	24.146			
	IT3	0.745	0.037	22.803			
	IT2	0.717	0.04	21.412			
Job alternative (JA)	JA2	0.915			0.941	0.941	0.841
	JA3	0.962	0.026	40.31			
	JA1	0.872	0.029	32.348			
Turnover intention (TI)	TI2	0.964			0.831	0.846	0.652
	TI3	0.703	0.047	18.442			
	TI1	0.731	0.046	19.294			

SE, Standard Error; CR, Critical Ratio; α, Cronbach’s Alpha; CR, Composite Reliability; and AVE, Average Variance Extracted.

Using SPSS to test the Cronbach’s Alpha reliability, the outcomes (see Table 1) revealed that each factor had a Cronbach’s Alpha above the 0.70 thresholds (0.831–0.941). These results imply that the scales used to assess the various constructs had good internal consistency.

The study further checked the validity of the constructs with composite reliability (CR) and average variance extracted (AVE) with the help of the AMOS plugin developed by Gaskin and Lim (2016). This AMOS plugin could automatically generate CR, AVE, discriminant validity (DV), and the correlation table using standardized coefficients and correlation values.

As seen in Table 1, all the CR values exceeded the 0.70 cutoffs (0.846–0.941), and those of AVE were higher than the 0.50 thresholds (0.594–0.841), as suggested by Joreskog and Sorbom (1993). These results show that the constructs had good convergent validity. Moreover, the discriminant validity values in bold along the diagonal path of the latent factor correlation matrix were greater than their corresponding inter-factor correlation coefficients (see Table 2). The DV results show that although the variables are related, they are unique and distinct from each other.

**Table 2:** Inter-factor correlation analysis.

	PJ	IF	JE	DJ	IT	JA	TI
PJ	**0.794**						
IF	−0.185^***^	**0.795**					
JE	−0.153^***^	0.383^***^	**0.771**				
DJ	0.061	−0.403^***^	−0.395^***^	**0.889**			
IT	0.237^***^	−0.308^***^	−0.359^***^	0.234^***^	**0.805**		
JA	0.003	−0.332^***^	−0.378^***^	0.329^***^	0.216^***^	**0.917**	
TI	0.218^***^	−0.344^***^	−0.467^***^	0.240^***^	0.364^***^	0.263^***^	**0.808**

*** *p *< 0.001.

The bolded values represent discriminant validity. PJ, Procedural justice; IF, Informational justice; JE, Job engagement; DJ, Distributive justice; IT, Interpersonal justice; JA, Job alternatives; and TI, Turnover intention.

Furthermore, a CFA model fit comparison test was done to establish the best model fit for the data set. As displayed in Table 3, the outcomes revealed that a 7-factor model fits best the data set in comparison to a 4-factor model and a 1-factor model, with Chi-square statistics (*X*^2^ = 498.299) to degrees of freedom (df = 413), normed Chi-square fit index (*X*^2^/df) = 1.207 (*X*^2^/df < 3.0 thresholds according to Hu and Bentler (1999)), comparative fit index (CFI) = 0.993, Tucker–Lewis fit index (TLI) = 0.992, and goodness of fit index (GFI) = 0.947 (CFI, TLI, GFI > 0.90 benchmark as suggested by Schuenemeyer (1989)). Likewise, the values of the standardized root mean square residual (SRMR) = 0.028, root mean square error of approximation (RMSEA) = 0.019 were within the acceptable levels, <0.08 and < 0.06, respectively (Hu and Bentler, 1999).

**Table 3:** Model fit comparison.

Models	*X* ^2^	*X*^2^/df	SRMR	RMSEA	CFI	TLI	GFI
7-Factor model	498.299	1.207	0.028	0.019	0.993	0.992	0.947
4-Factor model	548.675	1.294	0.046	0.023	0.990	0.989	0.942
1-Factor model	556.843	1.304	0.047	0.023	0.989	0.989	0.941

7-Factor model (Distributive justice, Procedural justice, Interpersonal justice, Informational justice, Job engagement, Perceived job alternatives; Turnover intention); 4-Factor model (4 organizational justice variables combined; Job engagement; Perceived job alternatives; Turnover intention); 1-Factor model (All variables combined); *X*^2^, Chi-square; *X*^2^/df, normed Chi-square fit index; SRMR, Standardized Root Mean Square Residual; RMSEA, Root Mean Square Error of Approximation; CFI, Comparative Fit Index; TLI, Tucker–Lewis Incremental Fit Index; GFI, the Goodness of Fit Index.

## Hypotheses testing

### Main effect and the mediating effect of job engagement

Using SPSS, hierarchical regression analysis helped estimate the main and mediating effects hypotheses, and the results are displayed in Table 4. The demographic characteristics such as gender, age, educational level, employment status, salary, work experience, and marital status were used as control variables. Model 2 in Table 4 shows the results of the effect of organizational justice on turnover intention. Distributive justice (*β* = 0.122, *p* < 0.010), procedural justice (*β* = 0.094, *p* < 0.050), and interpersonal justice (*β* = 0.276, *p* < 0.001) had a significant positive influence on frontline nurses’ turnover intention, making H1a, H1b, and H1c not supported. Contrariwise, informational justice (*β* = −0.189, *p* < 0.001) had a significant negative influence on nurses’ turnover intention; thus, H1d was supported. Likewise, Model 3 in Table 4 depicts the effect of organizational justice with all its subscales on job engagement. Distributive justice (*β* = −0.248, *p* < 0.001) and interpersonal justice (*β* = −0.226, *p* < 0.001) exerted a significant but negative effect on nurses’ job engagement, hence, H2a and H2c were not supported. However, procedural justice (*β* = 0.078, *p* < 0.050) and informational justice (*β* = 0.175, *p* < 0.001) both significantly and positively affected nurses’ job engagement, H2b and H2d were supported. Model 4 in Table 4 revealed that job engagement significantly and negatively predicted turnover intention (*β* = −0.434, *p* < 0.001), favoring H3.

**Table 4:** Hierarchical regression analysis results of the main effect and the mediating effect of job engagement.

Variables	Turnover intention	Turnover intention	Job engagement	Turnover intention	Turnover intention	Model 1 *β* (t)	Model 2 *β* (t)	Model 3 *β* (t)	Model 4 *β* (t)	Model 5 *β* (t)
(Constant)	5.187^***^ (7.744)	4.176^***^ (5.590)	4.597^***^ (6.897)	6.728^***^ (10.642)	5.532^***^ (7.366)
Gender	0.288 (1.015)	0.253 (0.972)	−0.427 (−1.838)	0.055 (0.210)	0.127 (0.504)
Age	0.006 (0.071)	0.037 (0.499)	0.017 (0.248)	0.030 (0.402)	0.042 (0.584)
Educational level	−0.005 (−0.038)	0.045 (0.373)	0.107 (0.996)	0.061 (0.504)	0.077 (0.658)
Employment status	−0.123 (−0.802)	−0.351^*^ (−2.474)	0.221 (1.748)	−0.122 (−0.867)	−0.286^*^ (−2.080)
Salary	−0.219^**^ (−2.811)	−0.216^**^ (−3.027)	0.072 (1.141)	−0.189^**^ (−2.642)	−0.194^**^ (−2.820)
Work experience	0.195 (1.721)	0.179 (1.719)	0.038 (0.409)	0.197 (1.893)	0.190 (1.892)
Marital status	−0.400 (−1.358)	−0.453 (−1.684)	0.437 (1.820)	−0.219 (−0.810)	−0.324 (−1.244)
DJ		0.122^**^ (2.773)	−0.248^***^ (−6.335)		0.048 (1.105)
PJ		0.094^*^ (2.345)	0.078^*^ (2.016)		−0.054 (−1.501)
IT		0.276^***^ (5.808)	−0.226^***^ (−5.337)		0.209^***^ (4.448)
IF		−0.189^***^ (−3.971)	0.175^***^ (4.111)		−0.137^**^ (−2.947)
JE				−0.434^***^ (−10.386)	−0.295^***^ (−6.478)
R^2^	0.021	0.190	0.236	0.178	0.246
∆ R^2^	0.021	0.169	0.221	0.156	0.225
F	1.753	12.044^***^	15.810^***^	15.304^***^	15.339^***^

β, Unstandardized estimates; (t), *t*-values; ^*^p < 0.05, ^**^p < 0.01, ^***^p < 0.001. PJ, Procedural justice; IF, Informational justice; JE, Job engagement; DJ, Distributive justice; IT, Interpersonal justice; JA, Job alternatives; and TI, Turnover intention.

Moreover, Model 5 in Table 4 presents the indirect impact of the different dimensions of organizational justice on turnover intention through job engagement. The outcomes highlighted that when job engagement is applied in the relationship between organizational justice and turnover intention, distributive justice, and procedural justice exert non-statistically significant impacts on turnover intention. However, job engagement (*β* = −0.295, *p* < 0.001) still significantly affected the turnover intention, implying that job engagement fully mediated those relationships. Hence, H4a and H4b were supported. On the other hand, interpersonal justice (*β* = 0.209, *p* < 0.001) and informational justice (*β* = −0.137, *p* < 0.010) had statistically significant influences on turnover intention in the presence of job engagement as a mediator. Since job engagement’s influence on turnover intention was still significant, this implied a partial mediation for job engagement in those relationships. H4c and H4d were also favorable.

### Moderating effect of perceived job alternative

Hierarchical regression analysis was also used with mean-centered variables (to avoid multicollinearity) to test the moderating effect of perceived job alternatives on the relationship between job engagement and turnover intention. From Model 2 in Table 5, it is still observed that job engagement significantly affected turnover intention negatively after being centralized, giving additional support for H3. Based on the estimation of Model 3 in Table 5, job engagement (*β* = −0.384, *p* < 0.001) and perceived job alternatives (*β* = 0.110, *p* < 0.010) had a negative and positive statistically significant influence on turnover intention, respectively. The same model also revealed that the interaction of job engagement and perceived job alternatives (*β* = 0.064, *p* < 0.050) was statistically significant. These outcomes suggest that perceived job alternatives moderated the relationship between job engagement and turnover intention, supporting H5.

**Table 5:** Hierarchical regression results of the moderating effect of perceived job alternative.

Variables	Turnover intention	Turnover intention	Turnover intention	Model 1 *β* (t)	Model 2 *β* (t)	Model 3 *β* (t)
(Constant)	5.187^***^ (7.744)	6.218 (9.434)	6.202^***^ (9.451)
Gender	0.288 (1.015)	0.026 (0.098)	0.043 (0.165)
Age	0.006 (0.071)	0.046 (0.616)	0.043 (0.572)
Educational Level	−0.005 (−0.038)	0.087 (0.720)	0.093 (0.770)
Employment Status	−0.123 (−0.802)	−0.137 (−0.977)	−0.128 (−0.918)
Salary	−0.219^**^ (−2.811)	−0.189^**^ (−2.662)	−0.193^**^ (−2.732)
Work Experience	0.195 (1.721)	0.178 (1.709)	0.171 (1.649)
Marital status	−0.400 (−1.358)	−0.211 (−0.784)	−0.214 (−0.797)
Job engagement		−0.395^***^ (−8.914)	−0.384^***^ (−8.672)
Job alternative		0.104^*^ (2.584)	0.110^**^ (2.739)
Job engagement x Job alternative			0.064^*^ (2.457)
R^2^	0.021	0.187	0.196
∆ R^2^	0.021	0.166	0.009
F	1.753	14.482^***^	13.754^***^

β, Unstandardized estimates; (t), *t*-values;

* *p *< 0.05;

** *p *< 0.01, and

*** *p* < 0.001.

## Discussion

This study was conducted under the challenging sanitary context of COVID-19. It aimed to establish the direct and indirect relationships between organizational justice and turnover intention through job engagement as a mediator. It was also a matter of testing the moderating effect of perceived job alternatives on the relationship between job engagement and turnover intention. The different scales were proven valid and reliable under the study’s conditions. Moreover, hierarchical regression analysis outcomes provided additional evidence for the suggested hypotheses.

### Effect of organizational justice on turnover intention

The results from the analysis indicated that three aspects of justice (distributive, procedural, and interpersonal) significantly and positively influenced turnover intention. These findings do not agree with our hypotheses and are inconsistent with most past research studies, where distributive justice (Akgunduz and Cin, 2015; Yang et al., 2021; Chen et al., 2022), procedural justice (Kumar, 2015; Gharbi et al., 2022), and interpersonal justice (Leineweber et al., 2020) were mainly observed to affect turnover intention negatively.

The implication could be that during the COVID-19 pandemic, Chinese frontline nurses felt injustice or unfairness in the distribution of outcomes, the procedure enactment, and interpersonal relationships at work, especially with their superiors. They could have perceived that their input was not rewarded accordingly, the decision-making procedures were inaccurate, and their relationships with their supervisors did not match their expectations.

Moreover, looking at the different distributive aspects (equity, equality, and need), the organizations might have applied equality instead of equity or need during their distribution of outcomes and decision processes, stipulating that being at the front line was not considered an extra investment that should be explicitly rewarded or what nurses would have liked was not necessarily needed. Contrariwise, the companies could have promoted esprit de corps and built group cohesion among workers by applying equality and dealing with available resources (Colquitt et al., 2005).

Interpersonal justice exacerbated turnover intention could also reveal that frontline nurses did not appreciate how their supervisors related to them. Instead of acting with courtesy and respect, they might have bruised their emotions, which could mitigate their level of acceptance of decisions and increase their intention to leave (Colquitt et al., 2013).

Only informational justice was found to affect turnover intention negatively, meaning that the way information was shared with frontline nurses did not automatically increase their desire to leave. This result aligns with prior findings (Suifan et al., 2017; Hussain and Khan, 2019). When it first appeared, COVID-19 was an unknown disease; information and procedures had to be updated frequently, sometimes daily, which was not easy to handle for frontline nurses (Sampaio et al., 2021). Moreover, the Chinese government marshaled important resources to control the pandemic successfully, and frontline nurses might have appreciated these efforts (Zhang Y. et al., 2020). Still, even if the information was misgiven or without manners, nurses might have understood that it was related to the uncommon and challenging situation and showed good attitudes toward their organizations.

### Effect of organizational justice on job engagement

In this study, distributive justice and interpersonal justice were found to significantly and negatively affect job engagement, while procedural justice and informational justice positively and significantly affected job engagement. These results are consistent with several empirical works that concluded a significant prediction of job engagement by organizational justice (Maslach et al., 2001; Saks, 2006; Ghosh et al., 2014; Pieters, 2018; Gupta et al., 2019; Suifan et al., 2020). Studies reported that distributive justice and procedural justice were positively related to job engagement (Pandey and David, 2013; Rasheed and Khan, 2013; Özer et al., 2017; Pakpahan et al., 2020), corroborating this study’s findings on procedural justice but contradicting the results concerning distributive justice. As for informational justice, this research’s outcome agrees with the works of Pakpahan et al. (2020) and Özer et al. (2017) but disagrees with those studies concerning interpersonal justice. Indeed, within a fair working environment, employees tend to be highly engaged and perform better (Suharti and Suliyanto, 2012). Employees’ participation in decision-making makes them feel valuable to the organization and enhances their engagement (Bin, 2016). Ghosh et al. (2014) contented that individuals’ satisfaction in allocating outcome procedures is not always related to their received outcomes. If they judged the distribution process fair and the products unfavorable, they can still boost their inner esteem and self-worth and respond with more engagement (Colquitt et al., 2005).

The pandemic context was not that appropriate for judging the procedures reasonably (Maben and Bridges, 2020; Mo et al., 2020; Zhang Y. et al., 2020). Because people’s lives were suddenly in danger, a quick response-action needed to be made, most of the decisions were taken from above, and nurses had to be executors (Cai et al., 2020; Hou et al., 2021). They probably could understand that, but still, they might not have been happy with the distribution of resources, outcomes, and the treatment manners of their supervisors. With stress, fear, and distress of the moment, they probably wished their superiors understood them more and acted with particular attention, kindness, and dignity and rewarded them accordingly.

### The direct and mediating effect of job engagement on turnover intention

The findings also revealed a negative and significant influence of job engagement on turnover intention, corroborating several similar results from previous research (Lu et al., 2016; Moo and Sung, 2017; Zhang et al., 2018; Cao et al., 2020; Zhang and Li, 2020). This prediction was strangely insignificant in Mi et al. (2020) work.

Moreover, job engagement significantly mediated the relationships between organizational justice and turnover intention, concurring with other studies (Al-Shbiel et al., 2018; Cao et al., 2020). Kaya et al. (2016) opined that good organizational practices enhance employees’ loyalty and belongingness to the company, reducing the intention to leave. Engaged workers will interact affectively with their work; regardless of the challenges, they will try not to lose motivation and show willingness and persistence to do their job well (Lu et al., 2016).

That says, during the COVID-19, frontline nurses showed a palpable dedication to their job and loyalty to the country and their organization by accepting to be at the front. The above finding suggests that while some organizational practices shook nurses’ engagement, they did not automatically expose a greater intention to leave.

### The moderating effect of perceived pob alternatives

**Figure 2:**
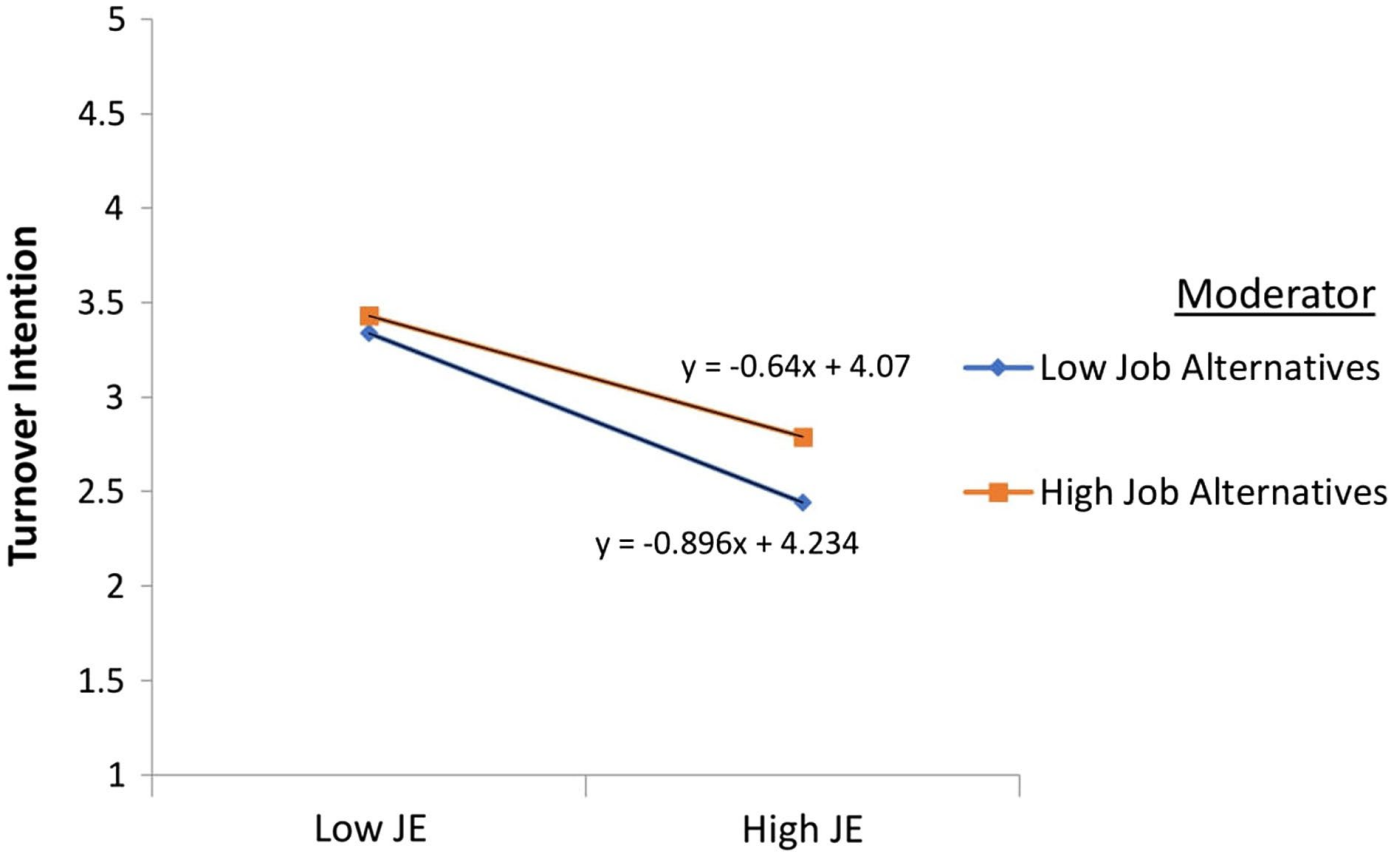
Moderating effect of perceived job alternatives on job engagement and turnover intention relationship.

The analysis’s introduction of perceived job alternatives exhibited interesting results and gave more insights concerning this variable’s influence on turnover intention. Findings highlighted that perceived job alternatives positively affected the leaving intent and moderated the relationship between job engagement and turnover intention. For instance, nurses’ likelihood of getting alternative jobs weakened that relationship, as shown in Figure 2. In this situation, employees could have a duality of affectivity and context. Though liking their career, extreme working conditions and the dangerous workplace could instigate their desire to continue in that profession, depending on the availability of alternative employment (Mushtaq et al., 2014). Contextually, the presence of COVID-19 not only downturned the economy but also increased unemployment, threatening nurses’ confidence in job availability (Song et al., 2020).

### Implications of the study

This study revealed theoretical and practical contributions in exploring how organizational justice can influence turnover intention in critical times. Theoretically, this work extends the literature on the variables involved in the conceptual framework, confirming the influencing strength of the causal variables, particularly in the nursing industry. First, in exploring organizational justice’s influence on turnover intention, this study has extended the knowledge on the specific aspects of organizational justice that significantly affect turnover intention. It has been demonstrated that interpersonal justice has the greatest predictive capacity on turnover intention, followed by informational justice, distributive justice, and procedural justice. Second, the integration of job engagement as a mediator supports the motivational role job resources should play to mitigate the adverse effects of job demands and foster the achievement of organizational goals (Schaufeli and Taris, 2014). In this study, job engagement has successfully mediated the relationship between the determinant variable (organizational justice) and the consequent variable (turnover intention), showing the nurses’ willingness to spend compensatory effort to attain their objectives. Therefore, the social exchange theory (SET) finds additional support in this study. Third, perceived job alternatives have been proven to weaken the relationship between job engagement and turnover intention. This supposes that the cognitive aspect of looking for alternative job opportunities is prompted in employees whose mental resilience, enthusiasm, and focus are challenged by limited resources, making them think of leaving (Hom et al., 2012; Mitchell et al., 2015). These findings reveal that inadequate treatments deteriorate the value of exchange and reciprocity expected in the workplace (Omar et al., 2018), giving additional explanation and support to the social exchange theory.

Practically, the study explicitly highlighted the justice aspects relevant to frontline nurses in critical times to enhance their staying intent. Some elements of organizational justice were found to exacerbate nurses’ engagement and turnover intent. The study’s outcomes encourage healthcare managers to provide frontline nurses with physical, psychological, and physiological resources to help them restrain job demands and associated costs (Bakker et al., 2007). Doing so will promote nurses’ personal growth, learning, and development and better equip them to attain their institutional aspirations (Moo and Sung, 2017; Scanlan and Still, 2019). As Yang et al. (2021) argued, the depletion of workers’ valuable resources does not favor their willingness to stick to the organization’s goals but instigates their intention to leave. The ability of perceived job alternatives to weaken the relationship between job engagement and turnover intention confirms frontline nurses’ unfavorable conditions and limited resources. Overall, it is suggested that good organizational practices should be promoted in medical companies to improve workers’ constructive attitudes and behaviors. Besides, increasing the perception of justice in the workplace can boost individuals’ engagement, making employees more enthusiastic about achieving the firm’s objectives. Such practices can also help curb leaving thoughts and active search for alternative employment opportunities, even in tough times.

### Limitations

Although this work had many contributions, it also presented some limitations. The cross-sectional nature of the study restraints it in a certain period. A longitudinal study may provide more substantial results of the hypothesized causal effects. Though the constructs of primary importance for this study showed significant influence on turnover intention, future studies may consider other personal and organizational variables such as work environment, routinization, negative affectivity, burnout, workplace violence, or comparison with the present job and explore their level of influence on nurses’ desire to stay in their profession. Moreover, the survey was conducted only in Jiangsu province, China, and among frontline nurses. Hence, generalizing the findings may not be appropriate. Since COVID-19 is still relevant in the whole country and the hypothesized relationships showed significant results, a similar approach can be tested nationwide, including more healthcare actors.

## Conclusion

This work showed that turnover intention among frontline nurses was exacerbated by poor implementation of distributive, procedural, and interpersonal justice. However, procedural and informational justice enhanced job engagement, while distributive and interpersonal justice negatively affected it. The intervention of job engagement as a mediator could mitigate the manifestations of the poor effects of distributive and procedural justice on nurses leaving intentions since it fully mediated those relationships. Still, the mediation was only partial for interpersonal and informational justice. Moreover, job engagement negatively influenced nurses’ turnover intention, and perceived job alternatives moderated that relationship by weakening its strength. This research’s findings and implications may give health policymakers and nursing managers insights into addressing the continual nursing shortage by controlling the turnover intention causing forces in critical times.

## Data availability statement

The data supporting the findings of this study are available from the corresponding author upon reasonable request.

## Ethics statement

The studies involving human participants were reviewed and approved by the Institutional Review Board of Jiangsu University, with the approval number JU-IRB: 05/08/21. The patients/participants provided their written informed consent to participate in this study.

## Author contributions

AK: conceptualization and writing—original draft preparation. PQ: methodology and formal analysis. TE: software and visualization. EN and SA-W: validation and data curation. EN: investigation. XX and AK: resources. TE and PQ: writing—review and editing. LZ: supervision. XX: project administration. LZ and XX: funding acquisition. All authors contributed to the article and approved the submitted version.

## Funding

This research was supported by the National Natural Science Foundation of China (grant no. 71974079) and the Social Science Foundation of Jiangsu Province (grant no. 20SHD002).

## Conflict of interest

The authors declare that the research was conducted in the absence of any commercial or financial relationships that could be construed as a potential conflict of interest.

## Publisher’s note

All claims expressed in this article are solely those of the authors and do not necessarily represent those of their affiliated organizations, or those of the publisher, the editors and the reviewers. Any product that may be evaluated in this article, or claim that may be made by its manufacturer, is not guaranteed or endorsed by the publisher.

## Appendix A

Questionnaire for the study.

**Table:**

Constructs	Description
Organizational Justice (OJ)	
1.Distributive Justice (DJ)	
DJ1	My reward reflects the effort I have put into my work
DJ2	My reward is appropriate for the work I have completed
DJ3	My reward reflects my contribution to the company
DJ4	My reward is justified, given my performance
2.Procedural Justice (PJ)	
PJ1	I am able to express my views and feelings at this company
PJ2	I feel I have influence over decisions at this company
PJ3	In general, procedures tend to be applied consistently
PJ4	Decisions that are made here are free of bias
PJ5	Decisions are based on accurate information
PJ6	Opportunities exist to appeal certain decisions
PJ7	Procedures comply with ethical and moral standards
3.Interpersonal Justice (IJ)	
	**Items stem: “My immediate supervisor….”**
IT1	Treats me in a polite manner
IT2	Treats me with dignity
IT3	Treats me with respect
IT4	Refrains from improper remarks or comments
4.Informational Justice (IF)	
	**Items stem: “My immediate supervisor….”**
IF1	Is open and frank in (his/her) communications with me
IF2	Explains the procedures thoroughly
IF3	Gives reasonable explanations regarding the procedures
IF4	Communicates details in a timely manner
IF5	Seems to tailor communications to individuals’ specific needs
Job Engagement (JE)	
JE1	I find the work that I do full of meaning and purpose
JE2	I am enthusiastic about my job
JE3	My job inspires me
JE4	At my work, I feel bursting with energy
JE5	I get carried away when I am working
Perceived Job Alternatives (JA)	
JA1	There are many available jobs similar to mine
JA2	I know of several job alternatives that I could apply for
JA3	I could easily find a better job than the one I have now
Turnover Intention (TI)	
TI1	I often think about quitting
TI2	Likely, ‘I will actively look for a new job next year
TI3	I will look for a new job next year
